# Contraceptive use among HIV-positive and negative women: implication to end unintended pregnancy

**DOI:** 10.1186/s40834-019-0084-2

**Published:** 2019-02-15

**Authors:** Amanual Getnet Mersha, Daniel Asfaw Erku, Sewunet Admasu Belachew, Asnakew Achaw Ayele, Begashaw Melaku Gebresillassie, Tadesse Melaku Abegaz

**Affiliations:** 1 Department of Gynecology and Obstetrics, School of Medicine, College of Medicine and Health Sciences, University of Gondar, Gondar, Ethiopia; 2 Department of Medicinal Chemistry, School of Pharmacy, College of Medicine and Health Sciences, University of Gondar, Gondar, Ethiopia; 3 Department of Clinical Pharmacy, School of Pharmacy, College of Medicine and Health Sciences, University of Gondar, Gondar, Ethiopia

**Keywords:** Contraceptive use, Ethiopia, HIV-positive women, Unintended pregnancy

## Abstract

**Background:**

With the advancement of antiretroviral therapy and improved life expectancy, women living with HIV/AIDS are enjoying a better sexual life. Yet, the consistent utilization of contraceptive in such patients is highly recommended. There is paucity of data regarding contraceptive use among HIV-positive and negative women in Ethiopia. The present study aimed at examining the use of contraceptives among HIV-positive and HIV-negative women in Ethiopia.

**Methods:**

A comparative cross-sectional study was conducted among HIV-positive and HIV-negative women attending family planning Clinic of Gondar university referral hospital between January 2016 and August 2017. Descriptive statistics were used to present categorical data and Pearson’s chi-square test was done to examine differences in the utilization of contraceptives between HIV-positive and HIV-negative women. Kaplan Meier test was also carried out to determine the incidence of unintended pregnancy. A *p*-value of 0.05 was deemed significant with corresponding 95% confidence intervals.

**Results:**

A total of 894 participants consisting of 314 HIV-positive and 580 HIV-negative women were included in the study. The rate of previous unintended pregnancy was 280 (31.3%) in HIV-negative women and 115 (12.9%) in HIV-infected women. Women who routinely utilized contraceptives were more likely to avoid unintended pregnancy [log rank: 2.89, *p* < 0.05]. Unlike HIV-negative women (2.9%), HIV-positive (28.4%) women reported a higher rate of intrauterine device use. Male condom was used more commonly in HIV-infected women (26.7%) as compared to HIV negative (3.9%) women *(p-value < 0.05).*

**Conclusions:**

Intrauterine contraceptive device was reported to be the most commonly used contraceptive method in HIV patients. Further, unintended pregnancy was relatively common in women with low contraceptive practice. The use of dual contraceptives should be advocated for HIV-positive women so as to protect unintended pregnancy and curtail the transmission of HIV.

## Background

Globally, 36.7 million people were living with HIV/AIDS at the end of 2016 and 50% of all adults with HIV infection were women [1]. Approximately, 70% of HIV infected individuals are located in sub-Saharan Africa. In Ethiopia, an estimate number of 1.1 million people are supposed to live with HIV at the end of 2010 and the prevalence is higher in females (1.9%) than males (1.2%) [2–5].

According to the 2016 Ethiopia demographic and health survey report, one in every three currently married women used a method of contraception mostly modern methods [2]. In addition, nearly one fourth of the current users (23%) were on injectable contraceptive method; 8% were using long acting or permanent methods; and, 4% of them were utilizing condom [2, 6].

HIV-infected women are at substantial risk of unintended pregnancy and other sexually transmitted infections (STIs). Hence, the recommended method of contraceptive is dual contraceptive method. Dual contraception is a utilization of one of the highly effective modern contraception coupled with condom to ensure protection from unintended pregnancy as well as STIs [9, 10].

A 2010 study conducted in Zambia reported the rate of dual contraceptive use to be 17.7% among all clients living with HIV/AIDS [7]. A comparative study conducted in Uganda reported that as compared to HIV negative individuals, sexually active HIV infected young counter people are less likely to use contraception and condoms [8] that make them vulnerable for other sexually transmitted infections, unintended pregnancy and its complications.

One of the most effective approaches to prevent mother to child HIV transmission is decreasing the rate of unintended pregnancy [9]. Irrespective of HIV status unintended pregnancy and unprotected sexual intercourse causes a major morbidity and mortality. In contrast to HIV none infected women, HIV infected women should consider multiple conditions including, the risk of having severe form of other sexually transmitted infections, resistant strains of HIV and greater risk of morbidity and mortality during pregnancy as well as motherhood period. Furthermore, HIV infected individuals are at risk of transmitting HIV to their uninfected partners. [10–12].

Irrespective of the HIV status of a woman, the inappropriate utilization of contraceptive leads to unintended pregnancy. All around the globe an estimated 80 million unintended pregnancies occur each year and the majority ended up in unsafe abortion [13, 14]. Unplanned pregnancy and unprotected sex may lead to many complications including but not limited to unsafe abortion, transmission of STIs, delayed or no prenatal care, poor maternal mental health, reduced quality of mother/child relationship, physical abuse and violence against women, poor developmental outcomes for children, increased risk of low birth weight as well as increased morbidity and mortality. The complications of unintended pregnancy were found to be significantly higher in HIV infected individuals [10–12].

There is paucity of data regarding the use of contraceptives among HIV-positive and negative women in Ethiopia. Evaluating current contraceptive use will enable clinicians to properly select and allocate appropriate contraceptive for both HIV infected and non-infected mothers. The current study aimed to determine the pattern of contraceptive use among HIV-positive and HIV-negative women. In addition, we intended to estimate unintended pregnancy in the set-up. It intends to help policymakers, service providers and program coordinators in Ethiopia to identify the difference that being HIV infected imposes on the choice of contraceptive methods as such evidence is an essential part to improve the reproductive health service delivery to HIV positive individuals.

## Methods

### Study design and setting

A comparative cross-sectional study was conducted to assess pattern of contraceptive use among HIV positive and HIV negative women attending family planning Clinic of Gondar university referral hospital (GURH), Northwest Ethiopia from January 2016 to August 2017. GURH is a teaching and referral hospital with more than 1000 beds and a range of specialties including internal medicine, pediatrics, surgery, gynecology, psychiatry, HIV/AIDS care and outpatient clinics.

### Study population and sampling technique

Sample size was determined using single population proportion formula considering the following assumptions: prevalence of unplanned pregnancy = 34% [21], confidence level of 95%, and a 5% degree of precision. Taking non-respondent rate = 10%, the final minimum sample size was 378. A convenience sample of HIV-negative and HIV-positive women who visited the family planning clinic of GURH during the study period was included in the study. Women who were unable to give the required information due to serious illness were excluded.

### Data collection procedure

Data was collected using an interviewer administered questionnaire. The data collection tool was created by modifying items from previously used instruments regarding contraceptive use among HIV-positive and HIV-negative women [7]. All items were thoroughly reviewed for relevance and content validity by a team of experts. The survey instrument was further validated by pre-testing on 20 participants who were not included in the final analysis. After the pretest, appropriate modifications were taken to the final questionnaire.

### Data analysis

Data were entered into and analyzed using Statistical Package for the Social Sciences (SPSS) software version 21.0 for windows. Descriptive data was presented as mean, standard deviation and percentages. Pearson’s chi-square test was used to examine the differences in contraceptive use between the study groups. Kaplan Meier test was carried out to examine the incidence of unintended pregnancy. A *p-*value of 0.05 was deemed significant with a corresponding 95% confidence interval (CI).

### Ethical considerations

This study was approved by the Institutional review board of University of Gondar. Informed written consent was obtained from each participant before starting the interview and participants were also told their right to stop the interview at anytime. The participants were also told that participation is based on their willingness. The obtained information was kept anonymous and recorded in such a way that the respondent could never be known.

## Results

### Socio demographic and clinical characteristics of participants

A total of 894 women were approached for the study that consist of 314 HIV-positive and 580 HIV-negative women. All of the invited women responded for the interview. The mean age of participants was 35 ± 3.21 years for HIV positive women and 33 ± 4.33 years for HIV-negative women. Nearly half of the respondents (49.7%) were urban residents and one third of the participants (32.5%) never went to school in both groups. Detailed socio-demographic characteristics of participants are presented in Table 1.

**Table 1:** Socio demographic characteristic of participants, (*n* = 894)

Variables	HIV Positive (*N* = 314)	HIV Negative (*N* = 580)	*P -* value
Age			0.56
Mean ± SD	35 ± 3.21	33 ± 4.33	
16–24	16(4.2%)	144(24.8%)	
25–34	143(45.5%)	258(44.4%)	
≥35	155(49.3%)	178(30.7%)	
Residency			0.36
Urban	156(49.7%)	281(48.5%)	
Rural	158(50.3%)	299(51.5%)	
Education			0.38
Never went to school	102(32.5%)	193(33.3%)	
Primary school	100(31.8%)	178(30.7%)	
High school	86(27.4%)	151(26%)	
College and above	26(8.3%)	58(10.1%)	
Employment status			0.27
Government employed	112(35.7%)	214(36.9%)	
Unemployed	202(64.3%)	366(63.1%)	
Monthly household income			0.67
< 500 birrs	8(2.5%)	133(22.9%)	
500–1000 birrs	198(63.1%)	364(62.7%)	
> 1000 birrs	108(34.4)	83(14.4)	
Mean ± SD	981 ± 102	952 ± 123	0.09
Marital status			0.81
Single	14(4.5%)	106(18.3%)	
Married	192(61.1%)	394(67.9%)	
Separated	52(16.6%)	54(9.3%)	
Widowed	56(17.8%)	26(4.5%)	

### Reproductive and clinical characteristics of participants

About 63.1% of HIV positive and 67.1% of HIV negative women reported to have only one sexual partner in the last 12 months period, and over a quarter of participants knew the sero-status of their partner. About 146(46.5%) of clients in the HIV infected group reported history of STI in the last 12 months as compared to 52(8.9%) with (*p* = 0.01, × ^2^ = 2.9) in non HIV infected women. Furthermore, 101(32.2%) of HIV positive women had partner history of STI as compared to 49(8.4%) in the HIV negative women. The rate of unintended pregnancy was 31.32% in HIV-negative women versus 12.86% in HIV-infected women (Table 2)**.**

**Table 2:** Reproductive and clinical characteristics of clients attending Gondar university hospital, *n* = 894

Variables	HIV Positive (*N* = 314)	HIV Negative (*N* = 580)	Chi-square test (× ^2^), *p- value*
Number of previous pregnancies			0.71, 4.93
None	19(6.1%)	41(7%)	
Once times	86(27.3%)	159(27.4%)	
Twice times	114(36.3%)	204(35.2%)	
Three or more times	95(30.2%)	176(30.3%)	
Pregnancy intention			0.04, ×^2^, 3.18
Intended pregnancy	199(63.4%)	300(33.6%)	
Unintended pregnancy	115 (36.6%)	280 (31.3%)	
Mean ± SD	2.4 ± 1.1	2.6 ± 1.3	0.4, ×^2^,6.75
Number of living children			0.60, ×^2^,1.49
None	26(8.3%)	44(7.6%)	
One child	92(29.3%)	172(29.6%)	
Two children	112(35.6%)	201(34.6%)	
Three or more children	84(26.7%)	163(28.1%)	
Mean ± SD	2.2 ± 1.2	2.1 ± 1.3	0.07, ×^2^,1.85
Desired number of children			
None	13(4.1%)	16(2.75)	
One child	65(20.7%)	93(16%)	
Two children	158(50.3%)	313(53.9%)	
Three or more children	78(24.8%)	158(27.2%)	
Mean ± SD	2 ± 1.1	2.4 ± 1.4	0.06, ×^2^,0.98
Number of sex partners in last 12 months			0.10, ×^2^,1.63
One	198(63.1%)	389(67.1%)	
Two	109(34.7%)	174(30%)	
Three or more	7(2.2)	17(2.9%)	
Mean ± SD	1.8 ± 1.3	1.6 ± 0.9	0.52, ×^2^,0.59
Menstrual irregularity			0.51, ×^2^,5.2
Yes	47(14.9%)	88(15.2%)	
No	267(85.1%)	492(84.8%)	
Chronic illness other than HIV/AIDS			0.93, ×^2^,2.6
Yes	19(6.1%)	19(3.3%)	
No	295(93.9%)	561(96.7%)	
Knowledge of partner’s HIV status			0.31, ×^2^,0.74
Yes	255(81.2%)	521(89.8%)	
No	59(18.8%)	59(10.2%)	
Desired number of childrenfulfilled			0.75, ×^2^, 1.8
Yes	205(65.3%)	434(74.8%)	
No	109(34.7%)	146(25.2%)	
Partner desires same number children			0.06, ×^2^, 1.3
Yes	202(64.3%)	437(75.3%)	
No	98(3.2%)	37(6.4%)	
Has no partner	14(4.5)	106(18.3%)	
Feeling if unexpectedly pregnant today			*P* < 0.05, X^2^,1.7
I would feel sad	223(71.1%)	192(33.1%)	
I would feel happy	64(20.4%)	294(50.7%)	
Indifferent	27(8.5%)	94(16.2%)	
History of STI within one year			0.01, X^2^ =2.9
Yes	146(46.5%)	52(8.9%)	
No	168(53.5%)	528(91.1%)	
Partner with previous STI			0.04, X^2^ =1.3
Yes	101(32.2%)	49(8.4%)	
No	213(67.8%)	531(91.6%)	

### HIV and HAART characteristics of HIV positive women

From a total of 314 HIV infected women, 153 (48.7%) were one HAART with a mean duration of HAART use of 10 ± 2.34 months. The mean duration of time since the diagnosis of HIV/AIDS was 2.7 ± 1.21 with a mean CD4 count of 692 ± 102.4 cells/m^3^. Approximately, three fourth 229 (73%) of women know the sero-status of their current partner and 233(74.2%) had disclosed their HIV status to their partner. Around 107 (34.1%) of clients had experienced stigmatization at least once in their life since acquisition of HIV (Table 3).

**Table 3:** HIV and HAART characteristics of HIV positive women (*n* = 314)

Variables	Frequency N (%)
HAART	
Yes	153(48.7%)
No	161(51.3%)
HIV status of current partner	
HIV Positive	229(73%)
HIV Negative	49(15.6%)
I don’t know	36(11.4%)
Time since HIV diagnosis years	2.7 ± 1.21
Duration of HAART use in months (Mean ± SD)	10 ± 2.34
Ever been stigma	
Stigmatized	207(65.9%)
Non- Stigmatized	107(34.1%)
Disclosed for the partner’	
Yes	233(74.2%)
No	81(25.8%)
CD4 count(mean ± SD)	692 ± 102.4 cells/m^3^

### Contraceptive method utilization

Intrauterine contraceptive device (IUCD) was reported to be used in 28.4% of HIV patients as compared to only 2.9% among HIV negative clients. Male condom was used more frequently in HIV patients (26.7%) as compared to HIV negative women (3.9%). Around 6.4% of HIV-positive women used dual contraceptive methods. Nearly a half of HIV-negative clients (50.1%) used injectable contraceptive method as compared to 25.8% of HIV positive women **(**Table 4).

**Table 4:** Types of contraceptive method used among HIV- positive and HIV-negative individuals

Method preference	HIV- Positive (294,93.63)	HIV-Negative (424, 73.1)	X^2^ -test, *p-value*
Oral contraceptive pills (OCP) of available type	22(7%)	48(12.1%)	2.6,p < 0.05
Injectable (Depot)	81(25.8%)	294(50.1%)	1.03, p < 0.05
Implant (Jadelle or Implanon)	16(5.1%)	40(6.9%)	4.01, p < 0.05
Intra uterine contraceptive device (IUCD)	89(28.4%)	17(2.9%)	1.9, p < 0.05
Consistent male condom use	84(26.7%)	23(3.9%)	2.8. p < 0.05
Consistent female condom use	2(0.6%)	2(0.3%)	2.7, p < 0.05
Dual contraceptive methods (condom and hormonal methods)	20(6.4%)	0(0%)	1.56, p < 0.05

### The effect of contraceptive use on the rate of unintended pregnancy

**Fig. 1:**
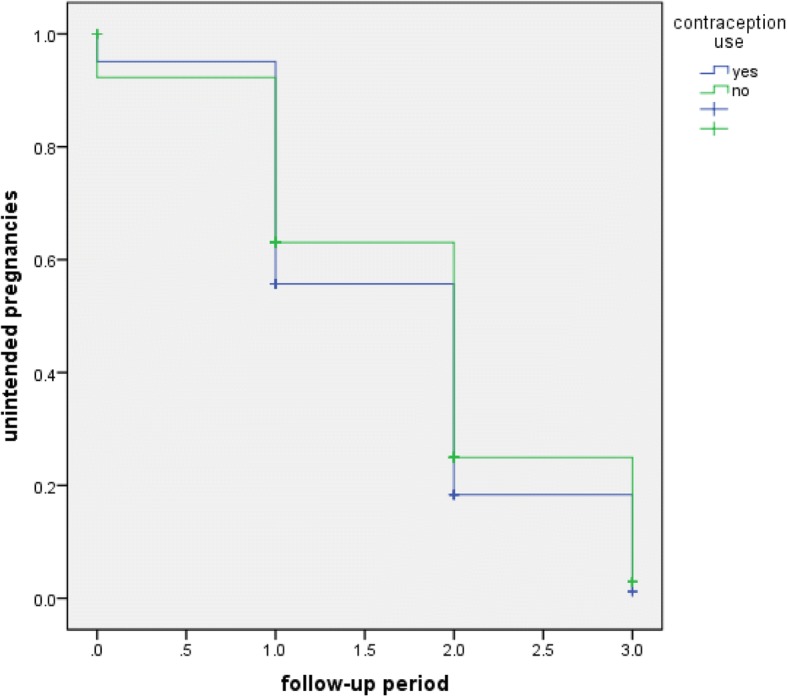
The effect of contraceptive use on the rate of unintended pregnancy

When women are followed for a mean period of three years for the trend of their past contraceptive use, those who routinely utilize contraceptive tended to avoid unintended pregnancy, [log rank: 2.89, *p - value* < 0.05].**(**Fig. 1).

## Discussion

Evaluation of contraceptive use among HIV sero-positive patients is essential so as to achieve effective control of unintended pregnancy and mother to child transmission of the retrovirus. Unintended pregnancy remained a major problem in developing nations particularly among HIV-positive women [4]. The current study aimed to compare the pattern of contraceptive use among HIV-positive and negative women.

In the present study, the use of at least one method of contraception was reported to be higher both in HIV infected 93.63% and HIV negative women (73.1%). However, previous studies reported low rate of contraceptive use in the same set-up. This discrepancy might be due to the changing attitude of clients towards family planning and the decline of risk of HIV transmission through interventions at antenatal care units and HIV follow-up clinics. Comparative study has been obtained from Addis Ababa, Ethiopia, in which about three-fourth of HIV infected women and nearly two-third of HIV non-infected women utilize contraceptive [15]. Contraceptive use was reported to be 34 and 59% among HIV-positive and negative individuals respectively among Ugandan population respectively [8]. Whereas, 42.6% of HIV -infected women were found to utilize a contraception method in Ghanaian society [16].

Our study revealed that, HIV positive patients usually utilized Intrauterine contraceptive device (IUCD) as compared to HIV negative clients who instead preferred hormonal contraceptives.. This might be due to the fact that hormonal contraceptives are expected to have untoward possible pharmacokinetic interactions between antiretroviral drugs which directs the preference to intrauterine device and condom use for HIV- positive clients on HAART [17]. In addition, the proportion of male condom use was significantly higher among HIV-positive women 84(26.7%) than HIV-negative ladies 23(3.9%). This finding is consistent with a study conducted in Malawi [18]. Condom is supposed to play dual purpose to protect both unintended pregnancy and STI. HIV-positive couples are advised to use this backup method as long as they decide to have sexual intercourse which by far increases the rate of utilization of condom as compared to HIV-negative mothers.

The utilization of dual contraceptive methods found to be frequently observed among HIV-positive patients. None of the respondents reported dual contraceptive use in the HIV non-infected group. The parallel application of condom use along with other methods is warranted to reduce the risk of unwanted pregnancy, transmission of HIV to a non-infected couple, to minimize transmission of drug resistant virus to an individual with HIV infection [19, 20]. Although, all HIV infected women are expected to use dual protection, the current study revealed that only limited number of patients (6.4%) applied dual contraception. Only small number of respondents (17.7%) reported dual method use in Zambia [7]. Poor utilization of dual contraception might contribute for higher incidence of STI in the sero-positive group 101(32.2%) versus 49(8.4%), in HIV negative clients. Further, under utilization of combination of methods could be due to high level of illiteracy 102(32.5%) of participants. A hospital based cross-sectional study also stated that low educational status was found to be associated with inadequate use of contraceptives among sexually active HIV positive individuals in the same set-up.

This study also illustrated that women who had unintended pregnancy used contraceptive methods occasionally. Even though Ethiopia has a promising achievement on contraceptive coverage, it is still difficult to control unintended pregnancies. This might be due to the lack persistence with the modern contraception. In addition, the low literacy and the high level of unemployment complicate the odds of unintended pregnancy in developing countries.

### Limitations

In general, the present study provided a comparative evidence regarding the pattern of contraceptive use among HIV-infected and non-infected women. However, its findings are only interpreted in the light of the following limitations. First of all the study is limited to single center and qualitative data on the reasons to choose a certain type of contraceptive is not included. Therefore, we recommend a multicenter study including large number of subjects and mixed type of study design to incorporate qualitative aspects. Further, the present study also highlighted that significant number of patients were not on HARRT. The reason for low consumption of the HARRT should be investigated.

## Conclusion

Intrauterine contraceptive device was reported to be the most commonly used contraceptive method in HIV patients as compared to HIV negative counter parts. Male condom use was found to be higher among HIV positive patients as compared to HIV negative women. Nearly, one-half of HIV negative clients use injectable contraceptive methods as compared to around a quarter in the HIV positive women. Further, unintended pregnancy was relatively common in women with low contraceptive practice. The use of dual contraceptives condom use in particular, should be advocated for HIV-positive women so as to protect unintended pregnancy, HIV transmission to partner, other strain and sub type HIV infections. Education and Communication campaigns should be strengthened in the community about reproductive health services in general and family planning services in particular. Access to quality contraceptive methods should be scaled up in community.

## Abbreviations

GURH: Gondar University Referral Hospital
HAART: Highly Active Antiretroviral Therapy
HIV: Human Immune Virus
IUCD: Intrauterine Contraceptive Device
STI: Sexually Transmitted Diseases
WHO: World Health Organization
